# Effectiveness of various human papillomavirus vaccination strategies: A community randomized trial in Finland

**DOI:** 10.1002/cam4.4299

**Published:** 2021-09-27

**Authors:** Matti Lehtinen, Dan Apter, Tiina Eriksson, Katja Harjula, Mari Hokkanen, Kari Natunen, Pekka Nieminen, Jorma Paavonen, Johanna Palmroth, Tiina Petäjä, Eero Pukkala, Simopekka Vänskä, Brigitte Cheuvart, Maaria Soila, Dan Bi, Frank Struyf

**Affiliations:** ^1^ Department of Vaccines National Institute for Health & Welfare Helsinki Finland; ^2^ Department of Laboratory Medicine Karolinska Institute Huddinge Sweden; ^3^ VL‐Medi Clinical Research Center Helsinki Finland; ^4^ Faculty of Social Sciences Tampere University Tampere Finland; ^5^ Department of Obstetrics and Gynecology University of Helsinki Helsinki Finland; ^6^ Finnish Institute for Health and Welfare Helsinki and Oulu Finland; ^7^ GSK Wavre Belgium; ^8^ GSK Espoo Finland; ^9^ GSK Wavre Belgium; ^10^ Present address: Janssen Research & Development Beerse Belgium

**Keywords:** community, gender‐neutral, human papillomavirus, vaccination strategy

## Abstract

**Introduction:**

We conducted a community‐randomized trial (NCTBLINDED) in Finland to assess gender‐neutral and girls‐only vaccination strategies with the AS04‐adjuvanted human papillomavirus (HPV)‐16/18 (AS04‐HPV‐16/18)vaccine.

**Methods:**

Girls and boys (12−15 years) were invited. We randomized 33 communities (1:1:1 ratio): Arm A: 90% of randomly selected girls and boys received AS04‐HPV‐16/18 vaccine and 10% received hepatitis B vaccine (HBV); Arm B: 90% of randomly selected girls received AS04‐HPV‐16/18 vaccine, 10% of girls received HBV, and all boys received HBV; Arm C: all participants received HBV. Effectiveness measurements against prevalence of HPV‐16/18 cervical infection were estimated in girls at 18.5 years. The main measures were: (1) overall effectiveness comparing Arms A or B, regardless of vaccination status, vs Arm C; (2) total effectiveness comparing AS04‐HPV‐16/18 vaccinated girls in pooled Arms A/B vs Arm C; (3) indirect effectiveness (herd effect) comparing girls receiving HBV or unvaccinated in Arm A vs Arm C. Co‐primary objectives were overall effectiveness following gender‐neutral or girls‐only vaccination.

**Results:**

Of 80,272 adolescents invited, 34,412 were enrolled. Overall effectiveness was 23.8% (95% confidence interval: −19.0, 51.1; *P* = 0.232) with gender‐neutral vaccination. Following girls‐only vaccination, overall effectiveness was 49.6% (20.1, 68.2; *P *= 0.004). Total effectiveness was over 90% regardless of vaccination strategy. No herd effect was found. Immunogenicity of the AS04‐HPV‐16/18 vaccine was high in both sexes.

**Conclusions:**

This study illustrates the difficulty in conducting community randomized trials. It is not plausible that vaccinating boys would reduce overall effectiveness, and the apparent lack of herd effect was unexpected given findings from other studies. This analysis was likely confounded by several factors but confirms the vaccine's high total effectiveness as in clinical trials.

## Graphical abstract

This community randomized trial evaluating two HPV vaccination strategies (girls only and gender neutral) in Finland failed to show indirect benefit for girls by vaccinating boys. Several factors might have prevented to show herd effect such as randomization bias. However, the study confirms the high total effectiveness of the vaccine in girls.

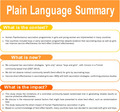

## 1 INTRODUCTION

Prophylactic vaccines against human papillomaviruses (HPVs) were first licensed in 2006 and 2007 for prevention of anogenital lesions. The first vaccines launched were the bivalent HPV‐16/18 vaccine adjuvanted with the Adjuvant System AS04 (AS04‐HPV‐16/18 vaccine) and the quadrivalent HPV‐6/11/16/18 vaccine, followed more recently by a nonvalent vaccine. Clinical trials have shown that the vaccines protect against the risk of HPV infection and cervical pre‐cancer in young women,^1^, ^2^ including cross‐protection against non‐vaccine HPV types.^3^, ^4^ Efficacy against anogenital HPV infections in men has also been demonstrated.^5^, ^6^

Organized HPV vaccination programs in girls and young women were implemented in many countries, primarily high‐income and upper‐middle‐income settings, shortly after the vaccines became available.^7^ A 2015 survey of European countries found that, of 27 countries, 16 had implemented girls‐only HPV vaccination programs, whilst 11 relied on opportunistic vaccination.^8^ Of the countries with the organized programs, most were delivered via school‐based vaccination. Few, if any, countries included boys in organized programs, despite modeling evidence of better vaccine effectiveness with gender‐neutral vaccination strategies.^9^

Prior to implementation of an organized vaccination program in Finland, we began a community‐randomized trial to evaluate the effectiveness of two vaccination strategies using the AS04‐HPV‐16/18 vaccine: gender‐neutral vaccination vs girls‐only vaccination.^10^ Data from the trial on vaccine safety and its effectiveness in reducing oropharyngeal HPV infections have been previously reported.^11^, ^12^ We now report the analyses of overall effectiveness measurements against cervical infections in vaccinated and unvaccinated adolescent girls.

**FIGURE 1:**
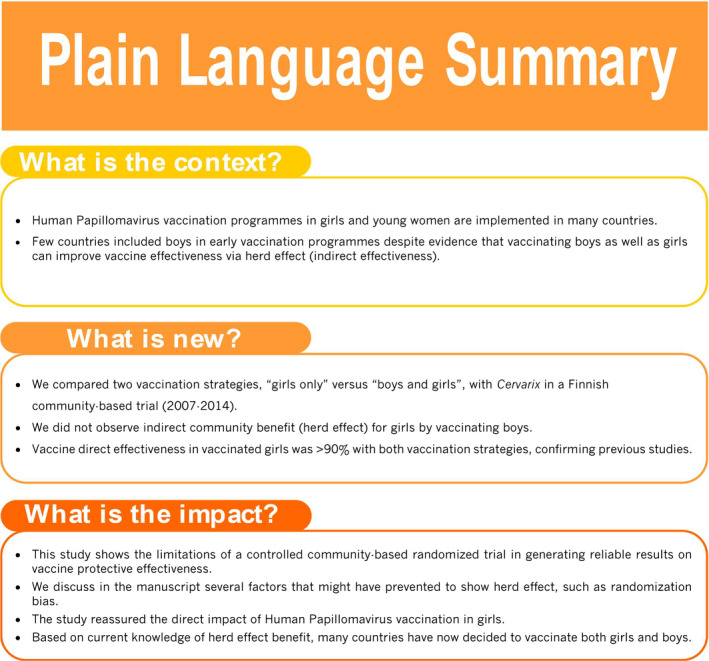
Plain language summary

Figure 1 summarizes the research, clinical relevance and impact of this study on the patient population.

## 2 METHODS

This phase III/IV, investigator‐initiated cluster randomized trial (NCTBLINDED) took place in Finland between October 2007 and December 2014. Its co‐primary objectives were to evaluate the overall effectiveness of two HPV vaccination strategies i.e., vaccinating both girls and boys (gender‐neutral), and vaccinating girls only.

The study was conducted in accordance with good clinical practice (GCP) and all applicable regulatory requirements including the Declaration of Helsinki, and was approved by the ethics committee of the Pirkanmaa hospital district in 2007 and 2011. Participants <15 years of age provided written informed assent and their parents or legal representatives provided written informed consent. In line with local regulations, participants ≥15 years of age provided written informed consent. Methodology and study characteristics have been published previously.^10^ The present paper reports immunogenicity and effectiveness of the vaccine against cervical HPV‐16/18 infection.

### 2.1 Community and participant eligibility

Communities were eligible to participate if they had 1000−9000 male and female adolescent inhabitants from the 1992−1995 birth cohort and were geographically distinct (at least 50 km apart or 35 km apart in the Helsinki metropolitan area). These minimum distances were imposed to minimize inter‐community transmission of HPV. We included 33 communities, stratified according to historical HPV‐16/18 seroprevalence rate in pregnant women under 23 years of age, estimated from the Finnish Maternity Cohort:^10^ (1) <20.5%, (2) 20.5%−24%, or (3) >24%. Twelve communities were included in stratum 1, nine in stratum 2, and 12 in stratum 3.

All male and female adolescents living in the study communities and born between 1992 and 1995 (12–15 years of age at the time of first vaccination)were eligible to participate. Girls had to be pre‐menarche or using adequate contraception before and during the vaccination period. Participants with acute disease at the time of enrollment and girls who were pregnant at the time of vaccination were excluded.

### 2.2 Study procedures

#### 2.2.1 Recruitment, randomization and masking

According to census information, 86,083 adolescents born between 1992 and 1995 resided in the 33 communities. Of these, we invited 80,272 native Finnish or Swedish speakers by letter to participate during two school years (2007−2008 and 2008−2009). Six thousand immigrants born in the same period who were non‐native Finnish or Swedish speakers were also eligible to participate, but were not invited by letter.

**FIGURE 2:**
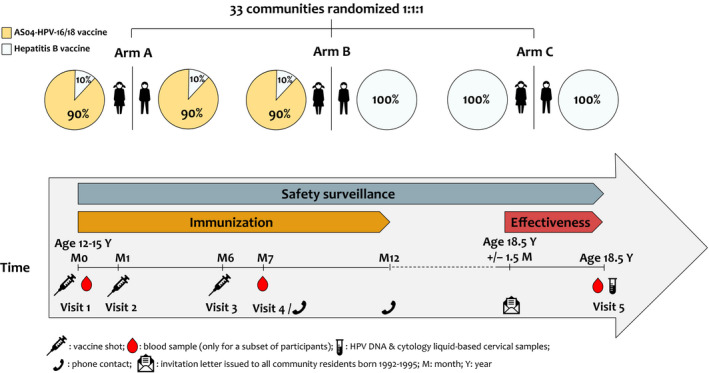
Study design

We randomized the 33 communities in a 1:1:1 ratio into three study arms: Arm A: 90% of randomly selected girls and boys received the AS04‐HPV‐16/18 vaccine and 10% of girls and boys received hepatitis B vaccine (HBV); Arm B: 90% of randomly selected girls received the AS04‐HPV‐16/18 vaccine, 10% of girls received HBV, and all boys received HBV; Arm C: all girls and boys received HBV (Figure 2). Randomization of communities was done by random number generation, stratified by historical HPV‐16/18 seroprevalence. Treatment allocation within each community in Arms A and B was performed at the investigator’s site using a central randomization system on internet (SBIR), stratified by community with a minimization procedure accounting for gender and birth year for all participants in Arm A, or by birth year only for girls in Arm B.

The study was open‐label for investigators. Participants knew which study arm their community was assigned to, but girls and boys in Arm A and girls in Arm B did not know whether they received AS04‐HPV‐16/18 or HBV. The study was open‐label for boys in Arm B and all participants in Arm C.

#### 2.2.2 Vaccination and follow‐up visits

The trial was divided into two phases: the immunization phase during which participants between 12 and 15 years of age (Visits 1−4), between Q4/2007 and Q1/2010, were vaccinated at all municipal junior high schools; and the effectiveness evaluation phase during which the impact of vaccination was evaluated in girls when they reached 18.5 years of age (Visit 5), between Q3/2010 and Q4/2014 (Figure 2).

During the immunization phase, school nurses administered the study vaccines intramuscularly in a three‐dose schedule (0, 1, and 6 months). Both the AS04‐HPV‐16/18 vaccine (*Cervarix*) and the HBV (*Engerix B*) were manufactured and supplied by GSK. AS04 is a GSK proprietary Adjuvant System containing 3‐*O*‐desacyl‐4′‐monophosphoryl lipid A (MPL; 50 µg) adsorbed on aluminum salt (500 µg Al^3+^). Blood samples were drawn from participants included in the immunogenicity subset at pre‐vaccination, at Month 7, and when participants reached 18.5 years of age (Visit 5). The immunogenicity subset was a convenience sample of boys and girls from pre‐selected communities in Arm A.

The effectiveness evaluation phase included participants previously enrolled in the vaccination phase and unvaccinated individuals joining the trial at Visit 5. We invited all girls and boys born between 1992 and 1995 in the 33 communities at 18.5 years of age, regardless of whether they had participated in the immunization phase. Girls attended the community study sites where a study nurse conducted pelvic examination and cervical sampling for HPV DNA testing. Girls and boys were asked to complete a questionnaire on community type and movement between communities, lifestyle, and sexual behavior. Crossover vaccination was offered i.e., AS04‐HPV‐16/18 vaccine offered to girls who had previously received HBV and vice versa. Boys who previously received HPV vaccine were offered HBV, but boys previously vaccinated with HBV could not be offered HPV vaccine because it was not indicated for boys at the time of the study end in 2014. Later, in 2017, crossover vaccination with HPV vaccine was offered also to these boys.

#### 2.2.3 Analysis of cervical samples and immunogenicity

A polymerase chain reaction (PCR) assay and multiplex type‐specific (MPTS) PCR Luminex assay were used to test cervical samples for DNA of 14 oncogenic types (HPV‐16, 18, 31, 33, 35, 39, 45, 51, 52, 56, 58, 59, 66, and 68) and 11 non‐oncogenic types (HPV‐6, 11, 34, 40, 42, 43, 44, 53, 54, 70, and 74), as previously described.^13^ The Bethesda 2001 system was used to report cervical cytology diagnoses. Histopathology was done on biopsy samples from girls with abnormal cytology referred for treatment and follow‐up.

Antibody responses against HPV‐16 and HPV‐18 were determined by enzyme‐linked immunosorbent assay (ELISA) in the immunogenicity subset. Seropositivity was defined as an antibody titer greater than the assay cut‐off value. To increase precision, at Visit 5, the assay cut‐off value was changed from 8 ELISA Units (EL.U)/ml to 19 EL.U/ml for HPV‐16 and from 7 EL.U/ml to 18 EL.U/ml for HPV‐18.

### 2.3 Study objectives

Several vaccine effectiveness measurements against the prevalence of HPV‐16/18 cervical infection were estimated in girls at 18.5 years of age (Table 1). Overall effectiveness compared the prevalence of infection in Arms A or B, regardless of vaccination status, vs Arm C. Total effectiveness compared the prevalence of infection in girls receiving AS04‐HPV‐16/18 vaccine in Arms A or B vs Arm C. This estimate included the direct effect of HPV vaccination plus an indirect effect arising from the expected vaccination‐related reduction in the circulation of HPV in the population of Arms A and B. Indirect effectiveness compared the prevalence of infection in girls receiving HBV or who were unvaccinated in Arm A vs Arm C (herd effect).

**TABLE 1:** Definition of vaccine effectiveness measures

Vaccine effectiveness measure	Endpoint^a^	Arms^b^ compared
Overall effectiveness in the invited cohort	Prevalence of infection in vaccinated and non‐vaccinated girls	A vs C (gender‐neutral strategy) B vs C (girls‐only strategy)
Total effectiveness in the enrolled cohort	Prevalence of infection in HPV vaccinated girls in arms A & B and in any enrolled girls in arm C Includes direct effect of vaccination plus an indirect effect arising from reduced HPV circulation in vaccinated populations	A vs C (gender‐neutral strategy) B vs C (girls‐only strategy) Pooled A/B vs C (regardless of vaccination strategy)
Indirect effectiveness in the enrolled cohort	Prevalence of infection in non‐HPV vaccinated girls (herd effect)	A vs C (gender‐neutral strategy)

Abbreviation: HPV, human papillomavirus.

^a^ HPV‐16/18 cervical infection in girls; non‐vaccinated girls included those who received control hepatitis B vaccine and those who received no vaccine.

^b^ Arm A: 90% of randomly selected girls and boys received the AS04‐HPV‐16/18 vaccine and 10% of girls and boys received hepatitis B vaccine; Arm B: 90% of randomly selected girls received the AS04‐HPV‐16/18 vaccine, 10% of girls received hepatitis B vaccine, and all boys received hepatitis B vaccine; Arm C: all girls and boys received hepatitis B vaccine.

The following confirmatory objectives were evaluated according to a hierarchical procedure (also referred to as a fixed sequence method in the context of multiplicity^14^): (1) to demonstrate overall vaccine effectiveness against HPV‐16/18 cervical infection in girls following vaccination of girls and boys (Arm A vs Arm C) (co‐primary objective); (2) to demonstrate overall vaccine effectiveness in girls against HPV‐16/18 cervical infection following vaccination of girls only (Arm B vs Arm C) (co‐primary objective); (3) to demonstrate total vaccine effectiveness against HPV‐16/18 oropharyngeal infection in girls (pooled Arms A and B vs Arm C, birth cohorts 1994 and 1995) (secondary objective, reported elsewhere^12^); and (4) to demonstrate indirect vaccine effectiveness (Arm A vs Arm C) against HPV‐16/18 cervical infection in the 1995 birth cohort (secondary objective).

Further secondary effectiveness objectives (exploratory) were to evaluate: (1) overall vaccine effectiveness against HPV‐16/18 cervical infection regardless of vaccination strategy (pooled Arms A and B vs Arm C); (2) total vaccine effectiveness against HPV‐16/18 cervical infection following vaccination of girls and boys, girls only, and regardless of vaccination strategy (Arms A, B, and pooled A/B, respectively, vs Arm C); (3) overall vaccine effectiveness against cervical infection with individual vaccine and non‐vaccine HPV types. Evaluation of immunogenicity of the AS04‐HPV‐16/18 vaccine was also a secondary objective.

### 2.4 Statistical analysis

#### 2.4.1 Determination of sample size

We expected to enroll on average 650 participants per community per year. Approximately 11 communities were required in each study arm to allow statistically powered evaluation of the two overall effectiveness confirmatory objectives (nominal power of 90% for each comparison) and the total effectiveness confirmatory objective (at least 80% power) under the assumptions of a two‐sided alpha of 0.05, vaccine coverage of 70%, HPV prevalence of 5.2%, 6.7%, and 12.6% in Arms A, B, and C, respectively, 15% coefficient of variation, and loss to follow‐up of 15%.

#### 2.4.2 Study cohorts

The invited cohort comprised all individuals born in the 33 communities between 1992 and 1995, and invited to participate in the trial. Effectiveness was evaluated from data available in the enrolled cohort, which comprised all study participants who enrolled, including those whose participation was limited to completion of a behavioral questionnaire. Only participants with measured endpoints were included for analysis of that endpoint. Immunogenicity was evaluated in the according‐to‐protocol cohort for immunogenicity, which comprised participants in the immunogenicity subset who met all eligibility criteria, complied with all procedures defined in the protocol (including receiving all doses of study vaccine), and had results available for antibodies against either HPV‐16 or HPV‐18.

#### 2.4.3 Effectiveness analysis

Vaccine effectiveness was computed as one minus the odds ratio of the prevalence of infection between the investigational arm(s) and the control arm. The analysis of overall effectiveness aimed to infer effectiveness in the invited cohort. Because the distribution of vaccination status (HPV/hepatitis B/no vaccination) in the invited cohort was different from the distribution in the enrolled cohort, the analysis of overall effectiveness used a weighted average of prevalence from unvaccinated and vaccinated female participants for the estimation of prevalence in the invited cohort in each arm. The weight was one for vaccinated female participants while, for unvaccinated female participants, it was the ratio of the percentage of evaluable participants (HPV DNA PCR cervical result available) among those receiving AS04‐HPV‐16/18 or HBV over the percentage of female individuals invited but not vaccinated from pooled Arms A, B, and C. No weighted average was used for the analysis of total or indirect effectiveness since these analyses were not based on a mix of AS04‐HPV‐16/18 vaccinated or unvaccinated participants.

The primary analysis of overall and total effectiveness was done using the Mantel Haenszel test adjusted for clustering and stratified by the historical seroprevalence used in the randomization. The 95% confidence interval (CI) on effectiveness and the two‐sided *P*‐value for the null hypothesis of no effectiveness were computed using the general inverse variance approach. The primary analysis of indirect effectiveness was done by logistic regression. The model included arm, birth cohort, and the interaction birth cohort × arm as fixed effects and community as random effect. Because it was expected that indirect effectiveness in Arm A would increase in each subsequent birth cohort as the size of the vaccinated male population increased, the demonstration of indirect effectiveness was based on a statistically significant effect in the 1995 birth cohort.

We used a hierarchical procedure to evaluate the multiple confirmatory objectives; objectives were evaluated in the order shown earlier. The first objective was met if the two‐sided *P*‐value based on the Mantel‐Haenszel test was <5%. The subsequent objectives were met if all previous objectives in the hierarchy had been reached and the associated two‐sided *P*‐value was <5%.

For the confirmatory objectives, the primary analysis was complemented by two sensitivity analyses: (1) Pearson chi‐squared test adjusted for clustering, without stratification; (2) multivariable logistic regression including community as random effect. The multivariable analysis was adjusted for the following covariates at the community level: seroprevalence used in the randomization, urban vs semi‐urban location, percentage of smokers at 15 years of age (from the behavioral questionnaire); and for the following covariates at the participant level: birth cohort (categorical variable for total and overall effectiveness and continuous variable for indirect effectiveness) and quarter of birth (Q1–Q2 vs Q3–Q4). We also did a post‐hoc analysis for the co‐primary objectives using a Mantel Haenszel test stratified by area type (urban vs semi‐urban) instead of by historical seroprevalence.

#### 2.4.4 Immunogenicity analysis

Seropositivity rates with exact 95% CIs and geometric mean titers (GMTs) with 95% CIs were calculated for HPV‐16 and HPV‐18. GMTs were calculated by taking the anti‐log of the mean of the log titer transformations. Antibody levels below the assay cut‐off value were assigned an arbitrary value of half the cut‐off for the purpose of the calculation of GMT.

## 3 RESULTS

**FIGURE 3:**
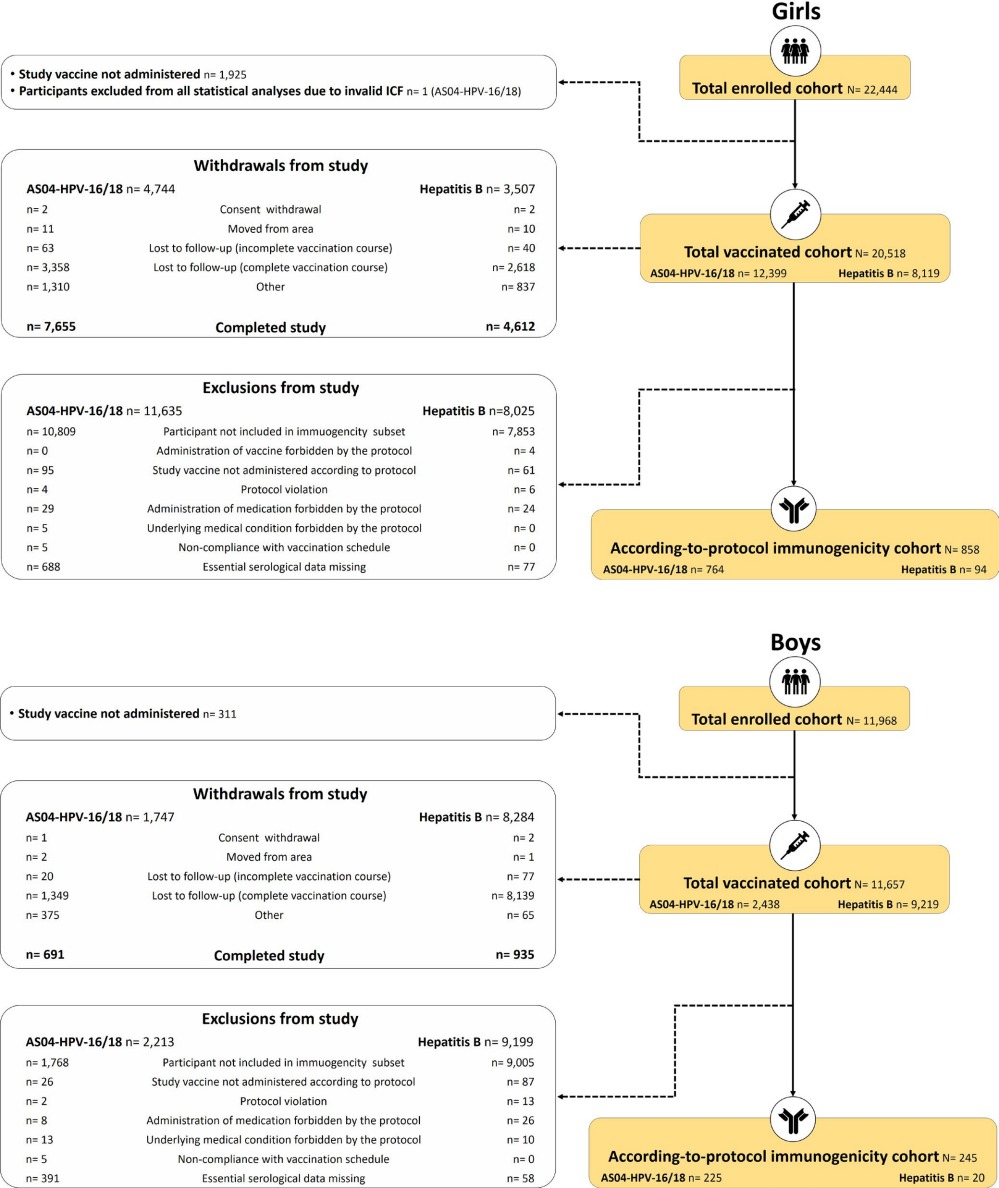
Participant disposition. AS04‐HPV‐16/18: AS04‐adjuvanted HPV‐16/18 vaccine; Hepatitis B: hepatitis B vaccine; ICF: inform consent form; N: number of participants; n: number of participants in a given category

In total, of the 80,272 adolescents invited, 34,412 were enrolled (enrolled cohort), including 22,444 girls and 11,968 boys^†^. A total of 1103 participants were included in the according‐to‐protocol immunogenicity cohort. Participant disposition through the study is shown in Figure 3. The mean age of the participants at first vaccination was 14.1 years and most participants were of White European origin (Table S1). AS04‐HPV‐16/18 vaccine coverage (i.e., the percentage of individuals invited to participate in the study who were vaccinated) was 47.0% for girls in Arm A, 45.7% for girls in Arm B, and 19.4% for boys in Arm A. Results of the behavioral questionnaire completed at 18.5 years showed that regular and weekend residency in the community, sexual behavior, as well as smoking, drinking, and drug consumption, were generally balanced between study arms (Table S2).

The prevalence of HPV‐16/18 in cervical samples from the AS04‐HPV‐16/18 vaccinated girls was 0.6% and 0.9% in Arms A and B, respectively (Table 2). In the hepatitis B and unvaccinated girls, the prevalence was 13.3% and 15.0%, respectively, in Arm A; 8.4% and 10.0% in Arm B; and 10.2% and 11.6% in Arm C (Table 2). Overall vaccine effectiveness against HPV‐16/18 cervical infection was 23.8% (95% CI: −19.0, 51.1; *P* = 0.232) following vaccination of girls and boys (Arm A vs Arm C; first confirmatory objective) and 49.6% (20.1, 68.2; *P* = 0.004) following vaccination of girls only (Arm B vs Arm C; second confirmatory objective) (Table 2). Indirect vaccine effectiveness against HPV‐16/18 cervical infection (Arm A vs Arm C) was −46.2% (95% CI: −118.4, 2.2; *P* = 0.064). As the first confirmatory objective was not met, in accordance with the hierarchical analysis, none of the other confirmatory objectives was considered to be met.

**TABLE 2:** Overall effectiveness of the AS04‐HPV‐16/18 vaccine against cervical infection with HPV‐16/18 in young women: vaccination of girls and boys (Arm A) or girls only (Arm B) (enrolled cohort)

Arm	N invited	Vaccine group	N	n (%)	Mantel Haenszel (confirmatory)^a^		Pearson chi‐square (sensitivity)^b^		Multivariable logistic regression (sensitivity)^c^		Mantel Haenszel (post‐hoc)^d^	
					Vaccine effectiveness, % (95% CI)	*P*‐value	Vaccine effectiveness, % (95% CI)	*P*‐value	Vaccine effectiveness, % (95% CI)	*P*‐value	Vaccine effectiveness, % (95% CI)	*P*‐value
A	12,243	AS04‐HPV‐16/18	2784	18 (0.6)	23.8 (−19.0, 51.1)	0.232	27.4 (−15.5, 54.4)	0.176	35.5 (6.0, 55.7)	0.022	29.7 (−0.0, 50.6)	0.050
		Hepatitis B	346	46 (13.3)								
		Unvaccinated	499	75 (15.0)								
		Total	3629	139 (8.1)								
B	14,570	AS04‐HPV‐16/18	3069	27 (0.9)	49.6 (20.1, 68.2)	0.004	50.1 (16.7, 70.2)	0.008	50.7 (33.0, 63.7)	<0.001	45.4 (11.8, 66.2)	0.013
		Hepatitis B	369	31 (8.4)								
		Unvaccinated	591	59 (10.0)								
		Total	4029	117 (5.7)								
C	12,607	Hepatitis B	2711	276 (10.2)	—	—	—	—	—	—		
		Unvaccinated	457	53 (11.6)								
		Total	3168	329 (10.9)								

Intra‐cluster correlation coefficient in Mantel Haenszel test: 0.0178 (Arm A vs C) and 0.0128 (Arm B vs C).

Weight used in estimate of prevalence: 5.5 (% evaluable participants among those receiving AS04‐HPV‐16/18 or hepatitis B vaccine over the % individuals invited but not vaccinated from pooled Arms A, B, and C: [9,279/20,519]/[1,547/18,901]).

Abbreviations: %: *n*/*N* except for the total, where %=(*n*[AS04‐HPV‐16/18]+n[hepatitis B]+w×n[not vaccinated])/(N[AS04‐HPV‐16/18]+N[hepatitis B]+w×N[not vaccinated]); AS04‐HPV‐16/18: AS04‐adjuvanted HPV‐16/18 vaccine; CI, confidence interval; Hepatitis B, hepatitis B vaccine; HPV, human papillomavirus; N invited, number invited to participate in the study; N, number of participants with available results; n, number of participants with HPV‐16/18 cervical infection; w, weight.

^a^ Adjusted for clustering and stratified by historical seroprevalence.

^b^ Adjusted for clustering without stratification.

^c^ Adjusted for covariates.

^d^ Adjusted for clustering and stratified by area type (urban or semi‐urban).

The results from the sensitivity analyses were consistent with the primary analysis, although the multivariable logistic regression estimated a higher overall vaccine effectiveness in Arm A than in the primary analysis (Table 2). The post‐hoc analysis with stratification by area type (urban or semi‐urban) instead of by historical seroprevalence estimated overall vaccine effectiveness of 29.7% (95% CI: −0.0, 50.6; *P* = 0.050) following vaccination of girls and boys, and 45.4% (11.8, 66.2; *P* = 0.013) following vaccination of girls only (Table 2).

**FIGURE 4:**
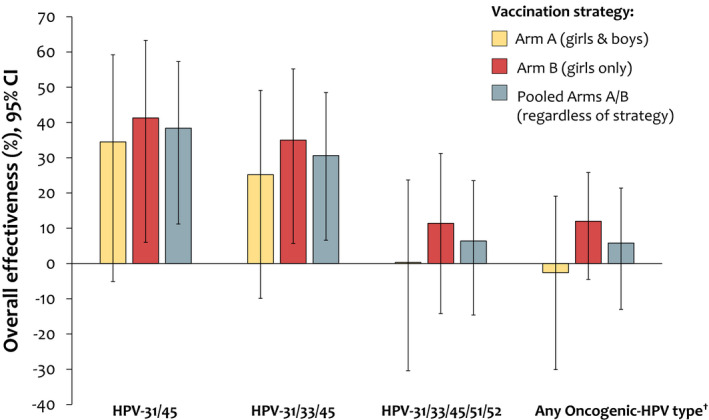
Overall effectiveness of the AS04‐HPV‐16/18 vaccine against cervical infection with different oncogenic HPV types in young women: vaccination of girls and boys (Arm A), girls only (Arm B), or regardless of vaccination strategy (pooled Arms A and B) (enrolled cohort). ^†^HPV‐16/18/31/33/35/39/45/51/52/56/58/59/66/68. Values are provided in Table S3. CI, confidence interval; HPV, human papillomavirus

For the exploratory objectives, overall vaccine effectiveness against cervical infection with HPV‐16/18 regardless of vaccination strategy (pooled Arms A and B vs Arm C) was 37.4% (95% CI: 9.6, 56.7; *P* = 0.013). We estimated total vaccine effectiveness of up to 93.8% following vaccination of girls and boys, girls only, and regardless of vaccination strategy (Arms A, B, and pooled A/B, respectively, vs Arm C) (Figure S1). In addition, we observed a similar range of overall vaccine effectiveness estimates against a composite of non‐vaccine HPV types (HPV‐31/33/45) regardless of vaccination strategy (Figure 4; Table S3).

**FIGURE 5:**
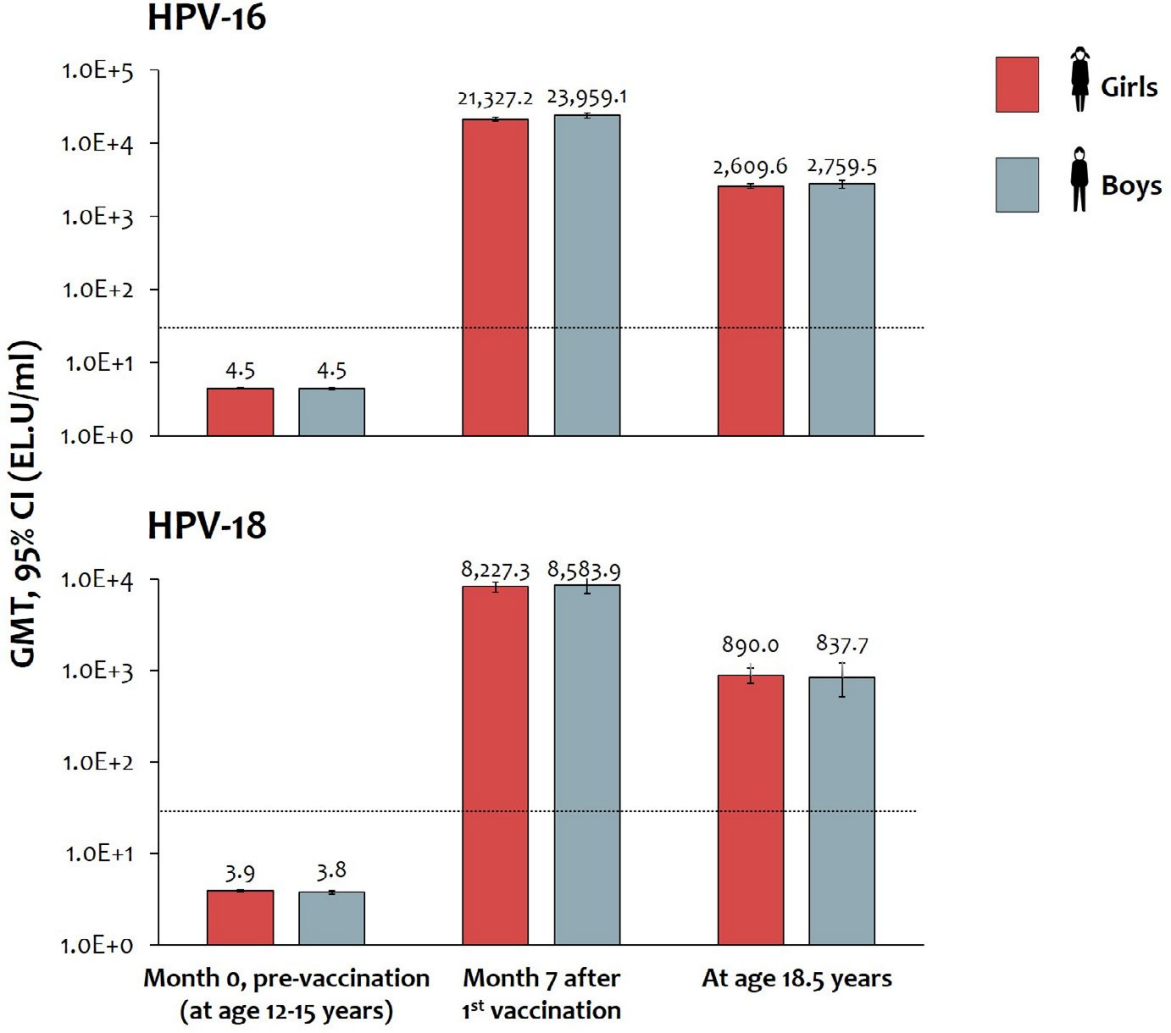
Immunogenicity of the AS04‐HPV‐16/18 vaccine in girls and boys (according‐to‐protocol cohort for immunogenicity). Data shown for all participants regardless of initial serostatus. GMT values shown above the bars. Dashed line shows the level of antibodies observed following clearance of a natural infection i.e., GMTs observed in women who were DNA‐negative and seropositive at baseline in a phase III efficacy study (29.8 EL.U/ml for HPV‐16 and 22.6 EL.U/ml for HPV‐18).^35^ CI, confidence interval; EL.U, ELISA units; GMT, geometric mean titer; HPV, human papillomavirus

Immunogenicity of the AS04‐HPV‐16/18 vaccine was similar in girls and boys (Figure 5). All initially seronegative girls and boys seroconverted following completion of the 3‐dose vaccination course, and antibody titers were sustained up to the final assessment at 18.5 years.

## 4 DISCUSSION

The study was designed to evaluate population‐based effectiveness with different HPV vaccination strategies. The first confirmatory objective of overall vaccine effectiveness against cervical HPV‐16/18 infection in young women following vaccination of girls and boys was not demonstrated with statistically significance (23.8% [95% CI: −19.0, 51.1]). Thus, none of the other confirmatory objectives could be considered to be met. However, we found evidence of overall vaccine effectiveness against cervical HPV‐16/18 infection following vaccination of girls only (49.6% [95% CI: 20.1, 68.2]). This was a counter‐intuitive finding, as there seems no plausible reason why including boys in the vaccination program would result in poorer vaccine effectiveness. Indeed, we had expected that overall effectiveness would have been higher in the per protocol analysis with a gender‐neutral vaccination strategy.

A possible explanation for the unexpected result was a failure of the randomization algorithm accounting for historical seroprevalence to allocate comparable communities to each study arm. Indeed, we observed almost 50% higher prevalence of HPV‐16/18 among unvaccinated young women in the study arm using gender‐neutral vaccination than in the girls‐only arm or the control arm. This does suggest a randomization bias that was detrimental to outcomes following gender‐neutral vaccination. We noted that area type (urban vs semi‐urban) appeared to be a better prognostic factor for HPV‐16/18 infection rates than the historical HPV‐16/18 seroprevalence. We therefore did a post‐hoc analysis using area type as a stratification factor in the analysis instead of historical seroprevalence. This analysis narrowed the gap between the overall vaccine effectiveness in the gender‐neutral arm vs the girls‐only arm, and CIs of the two effectiveness estimates overlapped considerably (29.7% [95% CI: −0.0, 50.6] vs 45.4% [11.8, 66.2]), respectively.

In addition, there might have been other assumptions that were incorrect and beyond our control, e.g., there might have been more interaction between the study population and other birth cohorts than was anticipated, in which case we would need to have vaccinated a broader cohort (across more birth years) than we did to see an effect between the arms. Since the study was open at the community level (participants knew which study arm their community was randomized to), there was a concern as to whether vaccine effectiveness could be affected by changes in sexual behaviors between different arms. However, there was no evidence of this in the data from the two behavioral questionnaires collected during the course of the study. Other studies have also reported that HPV vaccination does not result in increased sexual activity or risk‐taking sexual behavior among young men and women.^15^, ^16^, ^17^

The apparent lack of herd effect or benefit in vaccinating boys in the per protocol analysis was unexpected, given the wealth of evidence from real‐life studies and models demonstrating herd effect benefits of HPV vaccination.^9^, ^18^, ^19^, ^20^, ^21^, ^22^, ^23^, ^24^ A series of ancillary studies using the present study setting have evaluated gender‐neutral vaccination: (1) with regard to reduction in HPV prevalence from earlier to later birth cohorts (approximating a pre‐vaccination vs post‐vaccination comparison), (2) using cervico‐vaginal self‐samples with better compliance, increased sample size and balance between HBV‐vaccinated and non‐vaccinated referents and (3) adjusting for *Chlamydia trachomatis* prevalence,^25^, ^26^, ^27^ and finally (4) using an outlier‐free approach that excluded communities with exceptionally low (one arm B‐ and three arm A‐communities) or high vaccination coverage (one arm A‐community) according to an a priori plan.^27^ According to these ancillary studies the prevalence reduction within an arm (analysis 1) is not vulnerable by the randomization bias, the cervico‐vaginal self‐samples (analysis 2) probably provided more comprehensive data on the HPV prevalence than study nurse‐taken samples, and accounting for *C. trachomatis* prevalence (analysis 3) also helps in rectifying the randomization bias. These and the outlier‐free (analysis 4) analyses revealed a significantly stronger herd effect against HPV types 18/31/33 with the gender‐neutral vaccination strategy than with the girls‐only strategy.^27^ These ancillary studies were planned as an alternative to the per protocol analyses as early as 2011 while the clinical study was still ongoing. Most notably, results of the ancillary studies were recently confirmed by demonstrating HPV16 and HPV18 herd effects as significantly reduced post‐vaccination HPV16 and HPV18 seroprevalence among unvaccinated women of a similar age (under 23 years) in the gender‐neutral communities.^28^ Using the ancillary study data and parameters eradication of all hrHPV‐types, including HPV‐16 could be modeled with moderate coverage gender‐neutral strategy.^27^

We did identify substantial total vaccine effectiveness against HPV‐16/18 regardless of the vaccination strategy used. These findings are in line with those of clinical trials of the AS04‐HPV‐16/18 vaccine, which have shown similar levels of vaccine efficacy against HPV‐16/18 infection in young women, as well as protection against pre‐cancer endpoints.^29^, ^30^ Also in line with clinical trials,^3^ we found that the AS04‐HPV‐16/18 vaccine offered cross‐protection against non‐vaccine types HPV‐31/33/45. Cross‐protection against these HPV types has now also been shown in real‐world studies in several countries, including the UK, the Netherlands, Spain, and Japan.^21^, ^31^, ^32^, ^33^, ^34^ Furthermore, cross‐protection has been shown to contribute to high overall protection against high‐grade cervical intraepithelial neoplasia in young women in Scotland.^23^

At 18.5 years of age (3.5−6.5 years after first vaccination), anti‐HPV‐16 and anti‐HPV‐18 antibody titers remained above the level of antibodies observed following clearance of a natural infection in the PATRICIA study (i.e., GMTs observed in women who were DNA‐negative and seropositive at baseline).^35^ Immunogenicity of the vaccine was similar in males and females. Safety of the vaccine in this study has been reported elsewhere in detail, but no new safety concerns were identified and safety was similar in boys and girls.^11^

Strengths of the study have been described previously,^10^ and include the large sample size, 8‐year duration, uniform enrollment of girls and boys by school year, birth cohort, and community, and little movement between communities. HPV mass vaccination was introduced in Finland in 2013 for girls born from 1998, and there was therefore no confounding in our study from the national immunization plan. Local data indicated that opportunistic vaccination was also low. Both vaccinated and unvaccinated girls participated equally in cervical sampling at 18.5 years, and the study arms were similar with regard to sexual and behavioral characteristics, lifestyle, and mobility. Limitations included the likely failure of adequate randomization for HPV seroprevalence and lower vaccine coverage than expected, as already discussed.

To our knowledge, this is the only community randomized trial to evaluate different HPV vaccination strategies. Several countries have already implemented vaccination of boys based on modeling of indirect benefit to girls and potential direct benefit to boys for anal, penile, and oropharyngeal cancer. This study contributes a substantial amount of safety and immunogenicity data in boys vaccinated with the AS04‐HPV‐16/18 vaccine. However, even with this large sample size and the unique setting of Finland, with suitable infrastructure in place, we were unable to demonstrate overall effectiveness in the per protocol analysis. This illustrates the limitations of a controlled, community‐randomized trial for demonstrating indirect effectiveness. Such limitations were comprehensively explored and addressed using birth cohort and outlier‐free approaches in the ancillary study analyses.^25^, ^26^, ^27^ Further controlled studies evaluating different vaccination strategies will be impossible now that HPV vaccination programs are widely implemented.

In conclusion, conduct and interpretation of community randomized trial outcomes is not easy, and in this study was most likely confounded by factors such as different levels of baseline HPV prevalence between study arms despite randomization and the impact of urban vs semi‐urban residence. Thus, a direct comparison between the arms in this study did not reliably illustrate the potential of different vaccination strategies. Although the primary objective of the study was not met in the per protocol analysis, additional work done through the ancillary study and the independent community‐wise pre‐ and post‐vaccination HPV seroprevalence analyses has shed further light on the puzzling per protocol study results. The present study also confirms the high total effectiveness of the vaccine as indicated in clinical trials as well as its cross‐protective benefit, and adds important safety and immunogenicity data in boys and girls.

## CONFLICT OF INTEREST

The authors have no conflicts of interest to disclose.

## Supporting information

Supplementary Material

## Data Availability

Anonymized individual participant data and study documents can be requested for further research from www.clinicalstudydatarequest.com

## ENDNOTE

^†^
†The number of study participants shown is different to previously published data^10^ because an interim version of the study database was used formerly.
