# Supplementary material for: Effectiveness of various human papillomavirus vaccination strategies: A community randomized trial in Finland
Source: Cancer Med. 2021 Sep 27;10(21):7759–71. doi: 10.1002/cam4.4299 (PMC8559511; doi:10.1002/cam4.4299)
Supplement: Supplementary file 1 — Supplementary Material [file CAM4-10-7759-s001.pdf]

SUPPLEMENTARY FIGURE 1 Total effectiveness of the AS04-HPV-16/18 vaccine against cervical infection with HPV-16/18 in young women: vaccination of girls and boys, girls only, or regardless of vaccination strategy (enrolled cohort)

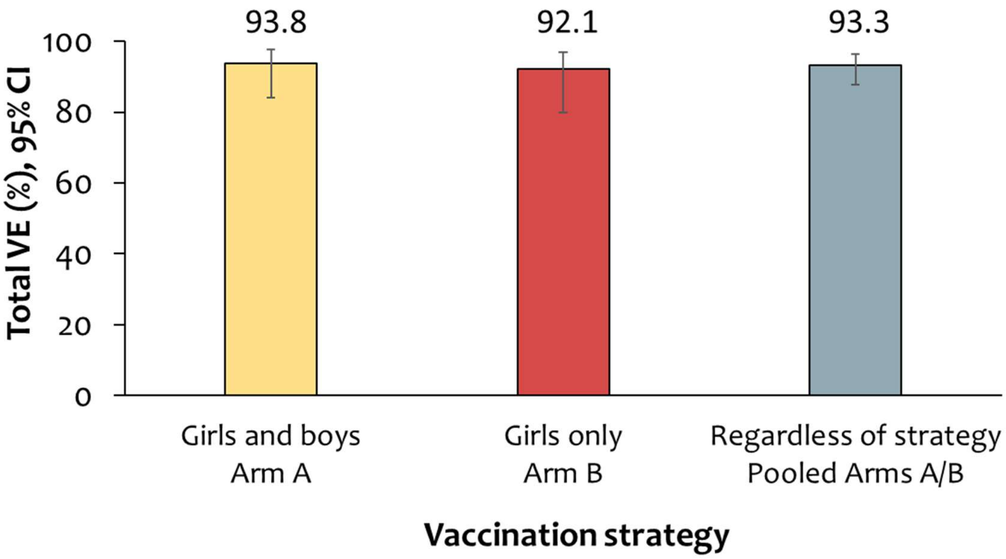

CI: confidence interval; HPV: human papillomavirus; VE: vaccine effectiveness

SUPPLEMENTARY TABLE 1 Participant demographic characteristics at baseline (enrolled cohort)

	Arm A						Arm B						Arm C			
	AS04-HPV-16/18		Hepatitis B		Not vaccinated		AS04-HPV-16/18		Hepatitis B		Not vaccinated		Hepatitis B		Not vaccinated	
	n or mean	%	n or mean	%	n or mean	%	n or mean	%	n or mean	%	n or mean	%	n or mean	%	n or mean	%
**Girls**	**N = 5,799**		**N = 669**		**N = 596**		**N = 6,601**		**N = 766**		**N = 732**		**N = 6,684**		**N = 597**	
Birth cohort																
1992	1,382	23.8	163	24.4	141	23.7	1738	26.3	204	26.6	171	23.4	1,754	26.2	153	25.6
1993	1,453	25.1	168	25.1	132	22.1	1656	25.1	192	25.1	166	22.7	1,552	23.2	143	24.0
1994	1,591	27.4	182	27.2	191	32.0	1660	25.1	191	24.9	208	28.4	1,732	25.9	158	26.5
1995	1,373	23.7	156	23.3	131	22.0	1547	23.4	179	23.4	187	25.5	1,646	24.6	143	24.0
Missing or NA	0	0.0	0	0.0	1	0.2	0	0.0	0	0.0	0	0.0	0	0.0	0	0.0
Birth quarter																
Q1−Q2	2,986	51.5	353	52.8	298	50.0	3,427	51.9	382	49.9	382	52.2	3,381	50.6	330	55.3
Q3−Q4	2,813	48.5	316	47.2	297	49.8	3,174	48.1	384	50.1	350	47.8	3,303	49.4	267	44.7
Missing or NA	0	0.0	0	0.0	1	0.2	0	0.0	0	0.0	0	0.0	0	0.0	0	0.0
Area type																
Urban	5,360	92.4	617	92.2	578	97.0	5,304	80.4	611	79.8	598	81.7	5,915	88.5	544	91.1
Semi-urban	439	7.6	52	7.8	18	3.0	1,297	19.6	155	20.2	134	18.3	769	11.5	53	8.9
HPV-16/18 seroprevalence stratum																
<20.5%	1,739	30.0	202	30.2	235	39.4	1,850	28.0	218	28.5	242	33.1	2,832	42.4	237	39.7
20.5−24%	1,418	24.5	162	24.2	153	25.7	1,344	20.4	159	20.8	139	19.0	1,358	20.3	106	17.8

	Arm A						Arm B						Arm C			
	AS04-HPV-16/18		Hepatitis B		Not vaccinated		AS04-HPV-16/18		Hepatitis B		Not vaccinated		Hepatitis B		Not vaccinated	
	n or mean	%	n or mean	%	n or mean	%	n or mean	%	n or mean	%	n or mean	%	n or mean	%	n or mean	%
>24%	2,642	45.6	305	45.6	208	34.9	3,407	51.6	389	50.8	351	48.0	2,494	37.3	254	42.5
Age at first vaccination, mean (SD), years	14.1 (0.75)	-	14.1 (0.76)	-	NA	-	14.1 (0.75)	-	14.1 (0.76)	-	NA	-	14.1 (0.75)	-	NA	-
Geographic ancestry																
White European	5,722	98.7	660	98.7	559	93.8	6,537	99.0	757	98.8	690	94.3	6,631	99.2	527	88.3
Other	77	1.3	9	1.3	12	2.0	64	1.0	9	1.2	5	0.7	53	0.8	6	1.0
Missing or NA	0	0.0	0	0.0	25	4.2	0	0.0	0	0.0	37	5.1	0	0.0	64	10.7
**Boys**	**N = 2,436**		**N = 299**		**N = 73**		**N = 2**		**N = 4,880**		**N = 129**		**N = 4,040**		**N = 109**	
Birth cohort																
1992	594	24.4	72	24.1	36	49.3	1	50.0	1,230	25.2	51	39.5	919	22.7	44	40.4
1993	546	22.4	71	23.7	17	23.3	0	0.0	1,234	25.3	41	31.8	948	23.5	35	32.1
1994	648	26.6	78	26.1	7	9.6	1	50.0	1,194	24.5	19	14.7	1,077	26.7	17	15.6
1995	648	26.6	78	26.1	13	17.8	0	0.0	1,222	25.0	18	14.0	1,096	27.1	13	11.9
Birth quarter																
Q1−Q2	1,289	52.9	151	50.5	46	63.0	1	50.0	2,498	51.2	62	48.1	2,057	50.9	60	55.0
Q3−Q4	1,147	47.1	148	49.5	27	37.0	1	50.0	2,382	48.8	67	51.9	1,983	49.1	49	45.0
Area type																
Urban	2,252	92.4	276	92.3	69	94.5	2	100	3,761	77.1	114	88.4	3,470	85.9	98	89.9
Semi-urban	184	7.6	23	7.7	4	5.5	0	0.0	1,119	22.9	15	11.6	570	14.1	11	10.1

	Arm A						Arm B						Arm C			
	AS04-HPV-16/18		Hepatitis B		Not vaccinated		AS04-HPV-16/18		Hepatitis B		Not vaccinated		Hepatitis B		Not vaccinated	
	n or mean	%	n or mean	%	n or mean	%	n or mean	%	n or mean	%	n or mean	%	n or mean	%	n or mean	%
HPV-16/18 seroprevalence stratum																
<20.5%	690	28.3	88	29.4	29	39.7	1	50.0	1,331	27.3	43	33.3	1,737	43.0	50	45.9
20.5−24%	621	25.5	76	25.4	10	13.7	1	50.0	1,137	23.3	24	18.6	880	21.8	14	12.8
>24%	1,125	46.2	135	45.2	34	46.6	0	0.0	2,412	49.4	62	48.1	1,423	35.2	45	41.3
Age at first vaccination, mean (SD), years	14.1 (0.78)	-	14.1 (0.76)	-	NA	-	14.0 (1.41)	-	14.1 (0.77)	-	NA	-	14.1 (0.76)	-	NA	-
Geographic ancestry																
White European	2,409	98.9	296	99.0	0	0.0	2	100	4,832	99.0	0	0.0	4,014	99.4	0	0.0
Other	27	1.1	3	1.0	0	0.0	0	0.0	48	1.0	0	0.0	26	0.6	0	0.0
Missing or NA	0	0.0	0	0.0	73	100	0	0.0	0	0.0	129	100	0	0.0	109	100

The number of study participants shown is different to previously published data because an interim version of the study database was used formerly (Lehtinen M, Apter D, Baussano I, et al. Characteristics of a cluster-randomized phase IV human papillomavirus vaccination effectiveness trial. *Vaccine*. 2015;33(10):1284-1290)

AS04-HPV-16/18: AS04-adjuvanted HPV-16/18 vaccine; Hepatitis B: Hepatitis B vaccine; HPV: human papillomavirus; N: number of participants; n: number of participants in a given category; NA: not applicable; SD, standard deviation.

SUPPLEMENTARY TABLE 2 Responses to behavioral questionnaire at Visit 5 (18.5 years of age) (enrolled cohort)

	Arm A				Arm B				Arm C			
	Girls N = 4,658		Boys N = 797		Girls N = 4,905		Boys N = 637		Girls N = 4,152		Boys N = 493	
	n or mean	%	n or mean	%	n or mean	%	n or mean	%	n or mean	%	n or mean	%
Usual residence												
Same community as school	3,961	85.7	711	90.5	4,258	87.3	560	88.7	3,500	84.8	442	90.4
Occasionally another community	190	4.1	29	3.7	174	3.6	17	2.7	163	3.9	15	3.1
Usually another community	65	1.4	5	0.6	61	1.3	9	1.4	63	1.5	2	0.4
Another community	406	8.8	41	5.2	387	7.9	45	7.1	403	9.8	30	6.1
Missing	36	-	11	-	25	-	6	-	23	-	4	-
Weekend and holiday residence												
Same community as school	3,129	67.4	574	72.3	3,380	69.1	462	72.8	2,722	65.7	352	72.1
Occasionally another community	1,431	30.8	207	26.1	1,426	29.2	162	25.5	1,351	32.6	130	26.6
Usually another community	85	1.8	13	1.6	84	1.7	11	1.7	73	1.8	6	1.2
Missing	13	-	3	-	15	-	2	-	6	-	5	-
Age at first menstruation, mean (SD), years	12.7 (1.3)	-	NA	-	12.6 (1.3)	-	NA	-	12.6 (1.3)	-	NA	-
Sexual debut at time of study visit												
Yes	3,648	78.5	554	69.8	3,892	79.5	391	61.6	3,258	78.8	298	60.7
No	1,000	21.5	240	30.2	1,005	20.5	244	38.4	878	21.2	193	39.3
Missing	10	-	3	-	8	-	2	-	16	-	2	-
Age at sexual debut, mean (SD), years	15.9 (1.3)	-	16.0 (1.3)	-	15.9 (1.3)	-	16.1 (1.3)	-	15.9 (1.3)	-	16.0 (1.4)	-
Number of new partners during the last year												
0	1,097	30.2	184	33.3	1,251	32.3	128	32.7	1,078	33.2	111	37.6

1	1,101	30.3	182	33.0	1155	29.8	153	39.1	948	29.2	105	35.6
2	563	15.5	86	15.6	574	14.8	44	11.3	491	15.1	38	12.9
3	386	10.6	36	6.5	371	9.6	22	5.6	331	10.2	19	6.4
4	220	6.0	35	6.3	254	6.6	23	5.9	167	5.1	10	3.4
≥5	270	7.4	29	5.3	272	7.0	21	5.4	230	7.1	12	4.1
Missing	1,021	-	245	-	1,028	-	246	-	907	-	198	-
Smoking												
Never smoked	2,719	58.6	489	62.0	3,049	62.4	460	72.4	2,569	62.1	344	69.9
Former smoker	318	6.9	49	6.2	352	7.2	32	5.0	316	7.6	26	5.3
Current cigarette smoker	1,586	34.2	228	28.9	1,473	30.2	130	20.5	1,247	30.2	109	22.2
Current smoker other than cigarettes	14	0.3	23	2.9	9	0.2	13	2.0	3	0.1	13	2.6
Missing	21	-	8	-	22	-	2	-	17	-	1	-
Alcohol consumption												
None	303	6.5	56	7.1	284	5.8	91	14.3	255	6.2	70	14.3
Once a month or less	1,765	38.0	257	32.4	1,820	37.2	202	31.8	1,603	38.7	142	29.0
2−4 times a month	2,196	47.3	364	45.9	2,374	48.6	256	40.3	2,003	48.4	202	41.2
1−2 times a week	355	7.6	104	13.1	391	8.0	77	12.1	267	6.5	69	14.1
Three times a week or more	22	0.5	12	1.5	18	0.4	10	1.6	11	0.3	7	1.4
Missing	17	-	4	-	18	-	1	-	13	-	3	-
Drug use												
Never	3,858	83.2	668	84.3	4,098	84.0	553	87.2	3,537	85.8	418	85.7
Once	379	8.2	52	6.6	386	7.9	34	5.4	301	7.3	33	6.8
Twice	153	3.3	30	3.8	139	2.8	17	2.7	121	2.9	14	2.9
Three times or more	248	5.3	42	5.3	257	5.3	30	4.7	165	4.0	23	4.7
Missing	20	-	5	-	25	-	3	-	28	-	5	-

N: number of participants; n: number of participants in a given category; NA: not applicable; SD: standard deviation.

SUPPLEMENTARY TABLE 3 Overall effectiveness of the AS04-HPV-16/18 vaccine against cervical infection with different oncogenic HPV types in young women: vaccination of girls and boys (Arm A), girls only (Arm B), or regardless of vaccination strategy (pooled Arms A and B) (enrolled cohort)

HPV type	Arm	N invited	Vaccine group	N	n (%)	Vaccine effectiveness, % (95% CI)	*P*-value
HPV-31/45	A	12,243	AS04-HPV-16/18	2,784	36 (1.3)	34.5 (-5.1, 59.2)	0.079
			Hepatitis B	346	31 (9.0)		
			Unvaccinated	499	30 (6.0)		
			Total	3,629	97 (4.0)		
	B	14,570	AS04-HPV-16/18	3,069	34 (1.1)	41.3 (6.0, 63.3)	0.027
			Hepatitis B	369	28 (7.6)		
			Unvaccinated	591	31 (5.2)		
			Total	4,029	93 (3.5)		
	Pooled A/B	26,813	AS04-HPV-16/18	5,853	70 (1.2)	38.4 (11.2, 57.3)	0.009
			Hepatitis B	715	59 (8.3)		
			Unvaccinated	1,090	61 (5.6)		
			Total	7,658	190 (3.7)		
	C	12,607	Hepatitis B	2,711	154 (5.7)	-	-
			Unvaccinated	457	28 (6.1)		
			Total	3,168	182 (5.9)		
HPV-31/33/45	A	12,243	AS04-HPV-16/18	2,784	85 (3.1)	25.2 (-9.9, 49.1)	0.139
			Hepatitis B	346	42 (12.1)		
			Unvaccinated	499	48 (9.6)		
			Total	3,629	175 (6.7)		
	B	14,570	AS04-HPV-16/18	3,069	88 (2.9)	35.0 (5.7, 55.2)	0.023
			Hepatitis B	369	36 (9.8)		
			Unvaccinated	591	49 (8.3)		
			Total	4,029	173 (5.9)		
	Pooled A/B	26813	AS04-HPV-16/18	5,853	173 (3.0)	30.6 (6.6, 48.5)	0.016
			Hepatitis B	715	78 (10.9)		
			Unvaccinated	1,090	97 (8.9)		
			Total	7,658	348 (6.3)		
	C	12,607	Hepatitis B	2,711	231 (8.5)	-	-
			Unvaccinated	457	40 (8.8)		
			Total	3,168	271 (8.6)		
HPV-31/33/45/51/52	A	12,243	AS04-HPV-16/18	2,784	380 (13.6)	0.3 (-3.4, 23.7)	0.985
			Hepatitis B	346	78 (22.5)		
			Unvaccinated	499	105 (21.0)		
			Total	3,629	563 (17.6)		
	B	14,570	AS04-HPV-16/18	3,069	412 (13.4)	11.4 (-14.2, 31.2)	0.351
			Hepatitis B	369	67 (18.2)		
			Unvaccinated	591	106 (17.9)		
			Total	4,029	585 (15.9)		

	Pooled A/B	26,813	AS04-HPV-16/18	5,853	792 (13.5)	6.4 (-14.6, 23.5)	0.523
			Hepatitis B	715	145 (20.3)		
			Unvaccinated	1,090	211 (19.4)		
			Total	7,658	1,148 (16.7)		
	C	12,607	Hepatitis B	2,711	445 (16.4)	-	-
			Unvaccinated	457	87 (19.0)		
			Total	3,168	532 (17.7)		
Any oncogenic HPV type†	A	12,243	AS04-HPV-16/18	2,784	675 (24.2)	-2.6 (-30.1, 19.1)	0.833
			Hepatitis B	346	117 (33.8)		
			Unvaccinated	499	173 (34.7)		
			Total	3,629	965 (29.7)		
	B	14,570	AS04-HPV-16/18	3,069	726 (23.7)	12.0 (-4.5, 25.8)	0.145
			Hepatitis B	369	103 (27.9)		
			Unvaccinated	591	170 (28.8)		
			Total	4,029	999 (26.4)		
	Pooled A/B	26,813	AS04-HPV-16/18	5,853	1401 (23.9)	5.8 (-13.0, 21.4)	0.519
			Hepatitis B	715	220 (30.8)		
			Unvaccinated	1,090	343 (31.5)		
			Total	7,658	1964 (27.9)		
	C	12,607	Hepatitis B	2,711	737 (27.2)	-	-
			Unvaccinated	457	146 (31.9)		
			Total	3,168	883 (29.5)		

†HPV-16/18/31/33/35/39/45/51/52/56/58/59/66/68

AS04-HPV-16/18: AS04-adjuvanted HPV-16/18 vaccine; CI: confidence interval; Hepatitis B: Hepatitis B vaccine; HPV: human papillomavirus; N invited: number invited to participate in the study; N: number of participants with available results; n: number of participants with HPV-16/18 cervical infection; %: n/N except for the total, where %=(n[AS04-HPV-16/18]+n[hepatitis B]+w*n[not vaccinated]/N[AS04-HPV-16/18]+N[hepatitis B]+w*N[not vaccinated]
